# One4Two^®^: An Integrated Molecular Approach to Optimize Infertile Couples’ Journey

**DOI:** 10.3390/genes12010060

**Published:** 2021-01-02

**Authors:** Valeria D’Argenio, Federica Cariati, Rossella Tomaiuolo

**Affiliations:** 1 Department of Human Sciences and Quality of Life Promotion, San Raffaele Open University, via di Val Cannuta 247, 00166 Roma, Italy; dargenio@ceinge.unina.com; 2 CEINGE-Biotecnologie Avanzate, via G. Salvatore 486, 80145 Naples, Italy; cariati@ceinge.unina.it; 3 Department of Molecular Medicine and Medical Biotechnologies, University of Naples Federico II, via S. Pansini 5, 80131 Naples, Italy

**Keywords:** infertility, diagnostic test, next generation sequencing, genetic test

## Abstract

The current diagnostic path of infertile couples is long lasting and often ineffective. Genetic tests, in particular, appear as a limiting step due to their jeopardized use on one side, and to the limited number of genes evaluated on the other. In this context, the development and diffusion, also in routine diagnostic settings, of next generation sequencing (NGS)-based methods for the analyses of several genes in multiple subjects at a time is improving the diagnostic sensitivity of molecular analyses. Thus, we developed One4Two^®^, a custom NGS panel to optimize the diagnostic journey of infertile couples. The panel validation was carried out in three steps analyzing a total of 83 subjects. Interestingly, all the previously identified variants were confirmed, assessing the analytic sensitivity of the method. Moreover, additional pathogenic variants have been identified underlying the diagnostic efficacy of the proposed method. One4Two^®^ allows the simultaneous analysis of infertility-related genes, disease-genes of common inherited diseases, and of polymorphisms related to therapy outcome. Thus, One4Two^®^ is able to improve the diagnostic journey of infertile couples by simplifying the whole process not only for patients, but also for laboratories and reproduction specialists moving toward an even more personalized medicine.

## 1. Introduction

Recent years have seen the progressive increase of knowledge in the field of reproductive medicine, due to the technological escalation that is making it possible to study the molecular bases of biological and pathological processes at a previously unthinkable resolution. Nowadays, the laboratory medicine applied to human reproduction allows several diagnostic [1,2] and screening options [3] with a positive impact on reproductive health [4].

Consequently, laboratory medicine is becoming an important player in the assessment of infertile couples as it can provide useful information to plan specific targeted interventions, as we have reviewed elsewhere [5].

Indeed, infertility incidence has enormously raised, affecting millions of couples worldwide (https://www.who.int/news-room/fact-sheets/detail/infertility). Thus, addressing and managing infertility is becoming an important issue for National Health Systems. It is now clear that the word “infertility” includes a complex and heterogeneous group of pathological conditions that may affect one or both partners and in which genes, lifestyles and environment are involved [5,6,7]. As a consequence, the diagnosis of couples’ infertility is a complex process that may require a too long duration. Molecular tests, in particular, are offered unevenly in different countries, and are often limited to a very small set of genes whose analyses may require different molecular techniques, so that they are commonly considered a bottleneck in the diagnostic path of infertile couples [8].

Based on the need of patients, reproduction specialists and laboratories to optimize the genetic tests, KronosDNA, a biomedical university spinoff active in the field of human reproduction [8], designed and developed One4Two^®^. 

One4Two^®^ is a customized next generation sequencing (NGS) panel that allows to simultaneously analyze: (1) genes related to genetic causes of both male and female infertility; (2) disease-genes of common hereditary diseases (cystic fibrosis, Duchenne’s dystrophy, hemophilia A and B and thalassemia); and (3) variants in genes coding for gonadotropins and their receptors and related to controlled ovarian stimulation outcomes. In addition, although the association between fertility and *BRCA* carrier status is still conflicting, the awareness about the possible risks of ovarian stimulation in *BRCA* mutations carriers and the possibility to perform preimplantation genetic testing to avoid the inheritance of the mutation, is increasing in both medical community and patients; thus, also the *BRCA* genes have been included in the panel in order to support reproduction specialists in the controlled ovarian stimulation [9].

**Figure 1:**
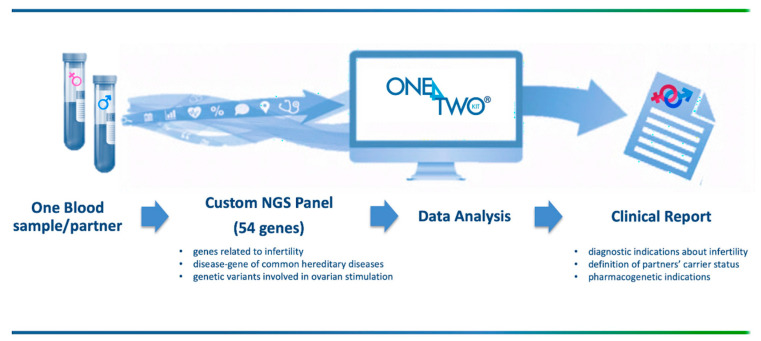
One4Two^®^ key points. This custom genes panel has been designed to simplify the diagnostic path of infertile couples: starting from just one blood sample/partner it allows the simultaneous analyses of a set of 54 genes providing data related to the genetic causes of infertility, partners’ carrier status of some of the most common inherited diseases, and also responsiveness to hormonal treatments. Indeed, patients have to undergo a single investigation, laboratories have to manage a single workflow, and reproduction specialists obtain relevant information in a single report tailored for each couple.

The comprehensive analysis offered by One4Two^®^ streamlines the workflow, brings together multiple diagnostic moments, and increases the clinically-actionable information (Figure 1).

Here, we describe the development and validation phases of One4Two^®^; moreover, using an approach based on the operation management, we characterize in detail its impact on the infertile couples’ journey.

## 2. Materials and Methods 

### 2.1. One4Two^®^ Genes Selection and Panel Design

**Figure 2:**
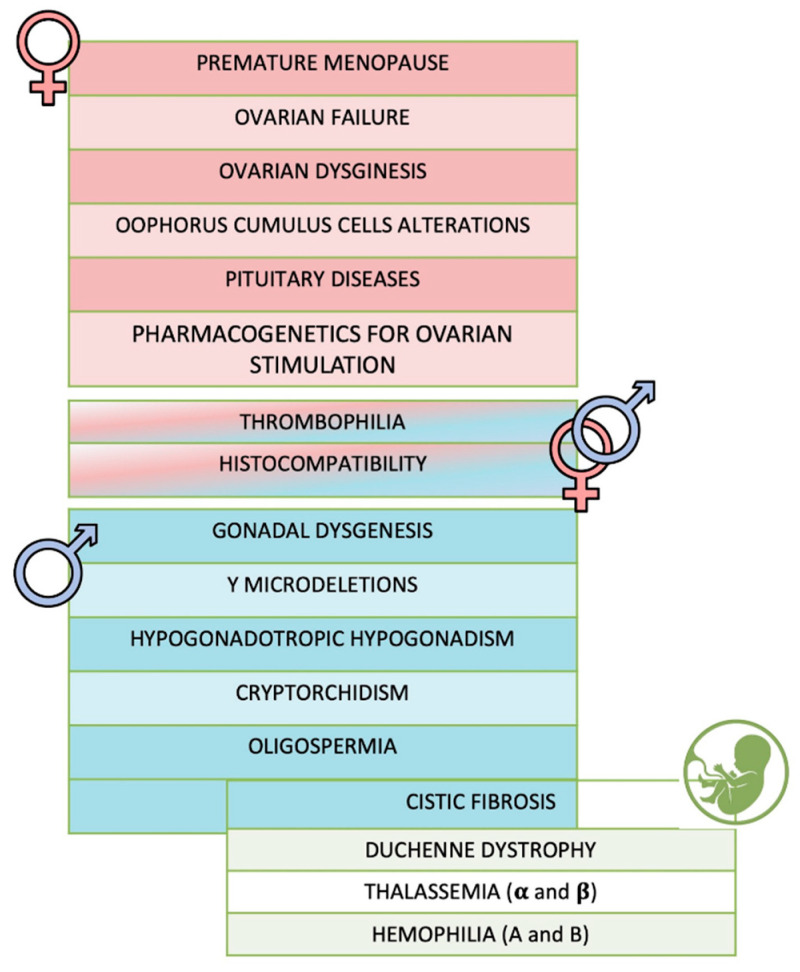
One4Two^®^ custom panel design. The final panel included 54 genes related to specific causes of both female and male infertility, and the causative genes of common inherited diseases.

The first step for the development of One4Two^®^ was the definition of the genes set to be included in the panel by an in-deep study of the literature. A total of 54 genes was selected, including 34 genes associated to female infertility, 22 genes to male infertility (10 genes being common between female and male), *BRCA1* and *BRCA2*, the causative genes of cystic fibrosis (*CFTR*), Duchenne dystrophy (*DMD*), hemophilia A (*F8*) and B (*F9*), and thalassemia (*HBB*, *HBA1* and *HBA2*) (Figure 2, Supplementary Table S1). The analysis of *FSHR*, *LHB* and *LHCGR* genes, included among the female ones, also allows identifying genetic variants involved in controlled ovarian stimulation.

For each gene, all the coding regions, 100 bp at 3’ and 5’ of each exon, the annotated promoters and 3’ UTRs were included in the design, obtaining a total target of 1 Mb.

### 2.2. Patients and Samples Selection

**Figure 3:**
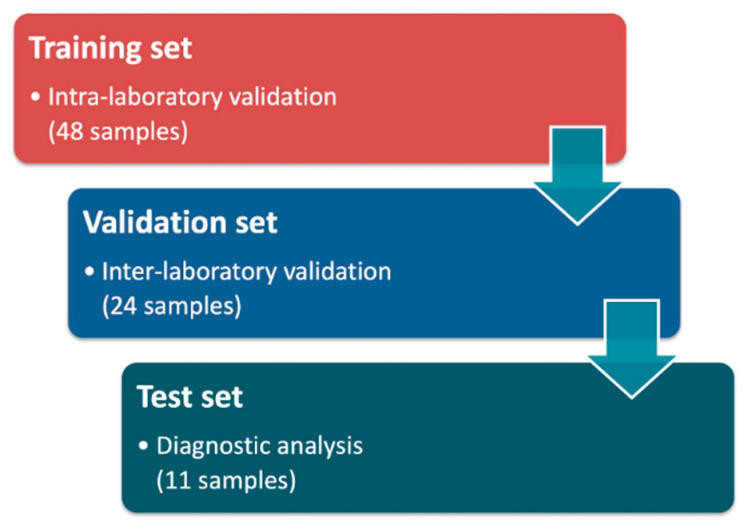
One4Two^®^ validation performance flow chart. The laboratory method performance was proven in two subsequent steps, intra and inter-laboratory validation, analyzing totally 72 genomic DNAs. Finally, a test set was performed on 11 subjects. In total, eighty-three samples have been analyzed herein.

The validation phase of the One4Two^®^ panel performances was carried out in three consequent stages, as summarized in Figure 3.

First, we performed an intra-laboratory validation by analyzing 48 subjects (training set, 24 females and 24 males) with previously identified variants in at least one of the genes included in our panel. Genomic DNAs extracted from peripheral blood samples were selected among those collected and stored, after molecular analyses by using traditional methods (reverse dot blot, HRM analysis and/or Sanger sequencing), in the biological samples bank of CEINGE Biotecnologie Avanzate (Napoli, Italy). When possible, we selected the DNAs of subjects who required a molecular test for causes related to infertility issues (such as patients requiring cystic fibrosis and/or thrombophilia molecular tests before to be admitted to an assisted reproductive technology intervention).

Then, we involved the Varelli Diagnostic Institute (Napoli, Italy), which provided a first set of 24 previously analyzed samples (validation set, 12 females and 12 males) and subsequently a second set of 11 not previously analyzed subjects (test set, 6 female and 5 male). The molecular analyses were conducted blindly. All the subjects analyzed in this study gave their written informed consent to the use of their DNA for research purposes anonymously.

### 2.3. DNA Libraries Preparation and Next Generation Sequencing

Genomic DNAs were obtained from a blood EDTA sample/patient. DNA extraction was carried out using the Maxwell 16 instrument (Promega, Madison, WI, USA). Next, the selected genomic DNAs were quantified using the Qubit picogreen assay (Life Technologies, Carlsbad, CA, USA) and their integrity was checked on the genomic screentape of the TapeStation (Agilent Technologies, Santa Clara, CA, USA). Libraries preparation has been carried out using the SureSelect QXT protocol, according to the manufacturer’s instruction. In detail, 50 ng of each DNA sample were enzymatically fragmented and specific adaptors were added to the ends of these fragments to univocally tag each sample. After a PCR amplification step, the DNA fragments were enriched by hybridization using the custom probes. Then, the enriched libraries were purified by using the AMPure XT beads (Beckman Coulter, Brea, CA, USA) and assessed for quality (TapeStation, Agilent Technologies, Santa Clara, CA, USA) and quantity (Qubit, Life Technologies, Carlsbad, CA, USA). Equimolar amounts of several libraries were pooled together to be simultaneously sequenced. Sequencing reactions were carried out on the MiSeq instrument (Illumina, San Diego, CA, USA) using the flowcell V2 PE 2X250. The libraries’ pool was loaded at a final concentration of 8pM with a 25% PhiX.

### 2.4. Bioinformatic Analysis and Clinical Report

FASTQ files were quality-filtered and aligned against the reference genome to obtain a VCF file/sample (Alissa Align and Call tool, Agilent, Santa Clara, CA, USA). Next, variants’ analysis and assignments were performed using the Expert Variant Interpreter (eVai) pipeline developed by enGenome (https://www.engenome.com, Pavia, Italy). The tool generates a list of variants/sample that are classified and prioritized according to international ACMG guidelines and a pathogenicity score [10]. 

Clinically relevant variants were selected to be included in a custom clinical report providing the presence of DNA variants related to infertility and/or to drug resistance or sensitivity, and the carrier status for the inherited diseases included in the panel.

### 2.5. Activities and Indexes to Evaluate the Infertile Couples’ Journey by An Operation Management Approach

The analysis of a process, by an operation management approach, requires the preliminary identification of the flow of actions. In the context of infertility diagnosis, we analyzed the physical-logistic path of infertile couples from the first medical evaluation to access to an assisted reproductive technology (ART). As a consequence, the entire process was disaggregated into its activities in a logical-temporal sequence and in order to highlight characterizing activities and bottlenecks. The flow was carried out based on the guidelines that formalize the diagnostic process of infertility, and on interviews with reproductive specialists and patients.

As key performance indicators (KPIs) were chosen the time that elapses from the first medical visit to the ART and the number of side activities.

## 3. Results

The One4Two^®^ NGS method was analytically validated by the retrospective analysis of 72 genomic DNAs into two different steps, the intra and inter-laboratory validations. Subsequently, the test set was performed on 11 subjects analyzed simultaneously with respect to the traditional lab approaches.

All the patients included in the present study were analyzed as described in Section 2, Materials and Methods. Up to 24 different, univocally tagged libraries were analyzed in a single MiSeq sequencing run ensuring a high sequencing depth and the coverage of the selected targets. Moreover, the simultaneous sequencing of 24 samples, being equivalent to 12 couples, is suitable with diagnostic laboratory turnaround-time in routine settings.

As the final aim was to validate our custom panel, the first step was to verify that we were able to detect all the variants previously identified in the same samples by traditional molecular methods. Both Training and Validation sets included patients attending a molecular diagnostic lab for cystic fibrosis and/or thrombophilia status assessment. Thus, we first analyzed *CFTR* and the thrombophilia genetic markers (*F2*, *F5*, *MTHFR*, *FXIIA1*, *FXIIIB*, *SERPIN1*, *FGB*, *ITGB3*, *AGT*, *ACE*, *APOE*) to verify the detected variants/sample. As expected, all the previously found DNA variants were identified also by One4Two^®^, thus confirming the analytic reliability of the proposed method.

Next, we aimed to extend the analysis to the whole genes panel in order to check for additional, clinically-relevant variants and to verify the increased diagnostic sensitivity of the proposed enlarged molecular test (Table 1).

Interestingly, we detected in three male patients a pathogenic variant in the *PROKR2* gene (c.518T>G, p.Leu173Arg) and in the *GNRHR* gene (c.317A>G, p.Gln106Arg), and a previously unreported variant in the *AR* gene. Moreover, 3 female patients had an *APOB* variant and one was found to be a carrier of a thalassemia-related variant (Table 1). Thus, also in this performance evaluation study, we were able to find additional genetic variants that would be lost by traditional approaches, assessing once again the diagnostic value of the method proposed herein.

After the intra- and inter-laboratory validation, we analyzed the 11 subjects included in the Test set. Five subjects were carriers of a *CFTR* mutation, one female subject carried the *HBB* pathogenic mutation c.118C>T (p.Gln40Ter) in heterozygous status, and all subjects were found to carry at least one thrombophilia-related variant. Additionally, in this case, the DNA variants identified by traditional diagnostic procedures were all detected also by One4Two^®^.

Finally, we evaluated the presence of some polymorphic variants that have been reported to be related to the ovarian stimulation outcome: rs6165, rs6166, and rs1394205 in the *FSHR* gene, rs1800447 and rs1056917 in the *LHB* gene, and rs229327 in the *LHCGR* gene [11]. As reported in Table 2, we are able to identify several women carrying these polymorphisms and classifiable with different ovarian stimulation outcomes, based on their genotypes [11].

### Operation Management of Infertile Couples’ Journey

**Figure 4:**
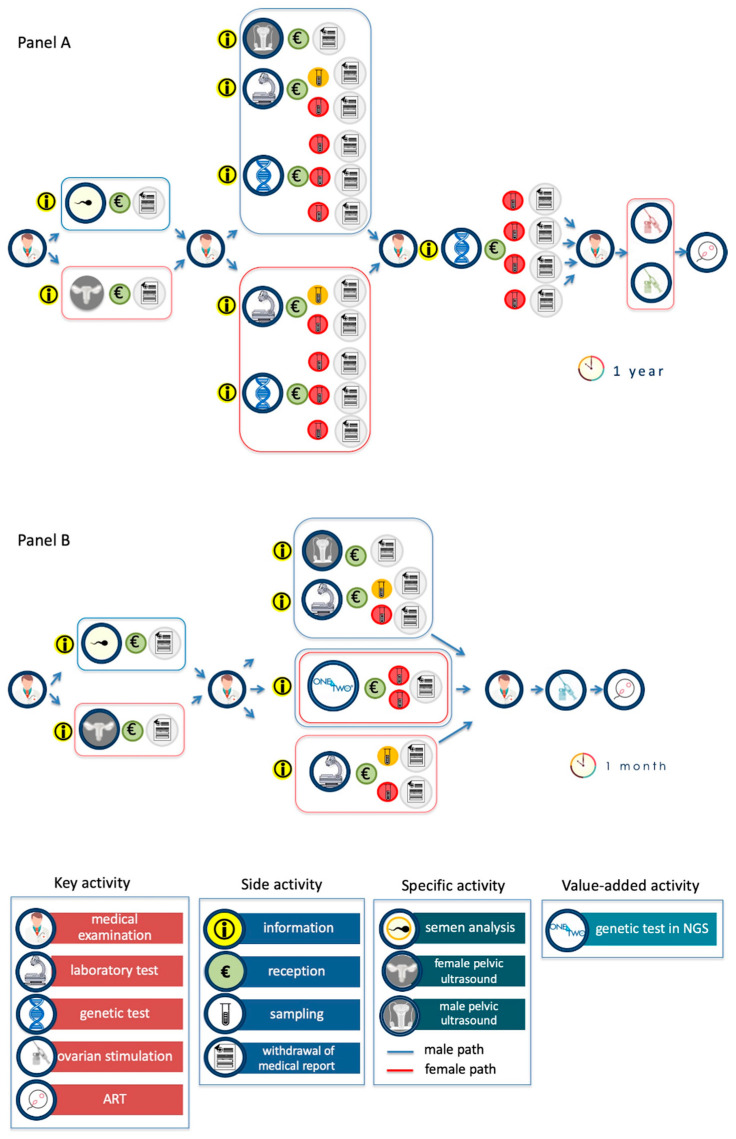
Comparison between infertile couples’ journey before (**A**) and after (**B**) the introduction of One4Two^®^. Some of the key operations, such as the three medical visits, the five traditional diagnostic investigations and the point of arrival (ART), remain unchanged with the introduction of One4Two^®^. However, the simplification of the key and side operations regarding molecular genetic diagnostics is evident. The execution of the test using NGS-technologies allows, not only to unify the analysis of genes related to infertility and that of disease-genes, but also to carry out the analysis simultaneously in both partners. Additionally, side actions are streamlined, as the collection of information and administrative practices are reduced from 8 to 6, the collection of biological samples is reduced from 17 to 8, and medical reports are reduced from 14 to 6. In (**B**) it is noted that the treatment for ovarian stimulation is beneficial, as the analysis of polymorphisms makes it possible to identify patients who are high or low responders to ovarian stimulation treatments. Finally, the time that elapses from the first medical visit to the ART goes from a year (**A**) to a month (**B**).

The current path of the infertile couple is described in Figure 4A. The key activities, i.e., the medical examination, the diagnostic investigations (execution of instrumental and/or laboratory tests), and genetic tests are highlighted. Diagnostic investigations include traditional laboratory tests (for example, hormonal profile assessment for women and semen analysis for men), genetic tests (for male are karyotype, analysis of Y microdeletions and of CFTR gene; for female are genes related to thrombophilia and to recurrent pregnancy loss) and also specific pelvic ultrasound. Each of these actions involves a series of repeated side actions (i.e., the collection of logistical information, booking, acceptance, execution of the investigation, waiting time, and, finally, the withdrawal of the report). This operation management analysis has allowed us to highlight the key and side activities, the decision-making steps and the bottlenecks of the whole, actually performed, process.

The key actions characterize the process and are perceived by patients as high value, i.e., the medical examination, the diagnostic investigation, the ovarian stimulation and the ART. Classically, the diagnostic path of infertile couples begins with the first medical visit and ends with ART. The whole process is characterized by the identification of the causes of infertility (diagnostic purpose) and by the definition of their reproductive partners’ carrier status (preventive purpose). The results of the first level diagnostic investigations (pelvic ultrasound for the woman and spermiogram for the man) constitute a decision-making step for the specialist and start the second level of investigations (for male pelvic ultrasound, karyotype, analysis of Y microdeletions and CFTR gene analysis; for female, evaluation of the hormonal profile, genetic tests for thrombophilia and recurrent pregnancy loss). Once the infertility diagnostic process is completed, the couple is required to carry out the reproductive partners’ carrier status for the identification of genetic pathologies that can be transmitted to the offspring.

The side activities are actions of low value for the patient, but necessary for the progression of the diagnostic flow, i.e., the collection of information required to identify the structures able to execute the diagnostic investigations, several required administrative procedures, the collection of the biological samples, and the withdrawal of the report. The main bottleneck is represented by the genetic tests, both for the execution times and for the difficulty in interpreting their results.

The diagnostic path of infertile couples with One4Two^®^ has been schematized in Figure 4B. One4Two^®^ has no impact on the key actions which do not require a molecular genetic diagnostic approach, such as medical visits, first-level diagnostic investigations and part of second-level investigations (pelvic ultrasound, karyotype for men; hormone profile assessment for women). While, from the analysis of the key performance indicators (KPIs), it is evident the positive impact in unifying genetic testing for diagnostic and preventive purposes, as there is a reduction in the time that elapses from the first medical visit to the ART (from a year to a month) and in the number of side activities. In addition, One4Two^®^ introduces high added value activities, such as the analysis of polymorphisms, that allow the reproduction specialists to perform personalized treatments for controlled ovarian stimulation.

## 4. Discussion

Overall, a significant percentage of infertility cases is due to genetic alterations. In particular, male infertility accounts for about 50% of cases of infertility, 15–30% of which being attributable to a known genetic factor [5]. Therefore, the diagnosis of infertility should arise from the combination of an accurate personal history of the patient and the couple, and instrumental and laboratory evaluations, including targeted genetic tests [5]. Despite this, genetic testing is not recommended for all patients, since it is an expensive and slow process, requiring several tests to analyze all the genes related to infertility.

NGS-based approaches allow the simultaneous interrogation of multiple pathogenic variants in many genes [12,13]; moreover, the combination of the test with an algorithm for data processing allows to streamline the analytical process [14]. Indeed, this approach is especially suitable for the study of complex diseases with variable phenotypic features related to a high genetic heterogeneity, like infertility [15]. There are valid reasons to consider introducing an NGS panel into the diagnostic pathway of infertile couples, provided that an appropriate selection of clinically relevant genes has been made [16]. However, it is important to remember that balanced translocations and complex chromosomal rearrangements cannot be detected using NGS [15]. 

Taking into account these needs, KronosDNA has developed One4Two^®^, an NGS-based genetic test with three purposes: the identification of the genetic causes of infertility, the definition of partners’ carrier status, and the analysis of clinically-relevant variants for personalized treatments. KronosDNA received positive feedback from both laboratories on streamlining workflow, and from reproduction specialists who showed satisfaction for the diagnostic efficacy [8].

The performance validation of One4Two^®^ gives positive results. First, the data obtained by One4Two^®^ are perfectly comparable with those obtained with traditional methods, without false positives nor false negatives results, thus providing 100% accuracy. 

Moreover, additional clinically-relevant variants have been also identified. Interestingly, we detected in a male patient a variant in the *PROKR2* gene (c.518T>G, p.Leu173Arg) (Table 1). Indeed, even if this variant has been reported as pathogenic, its clinical significance still appears conflicting. The *PROKR2* gene encodes for the prokineticin receptor 2, a G-protein coupled receptor, expressed on cell surface, and involved in the regulation of various cell functions. The p.Leu173Arg *PROKR2* variant, in both heterozygous or homozygous status, has been reported several times in individuals with Kallman syndrome or hypogonadotropic hypogonadism, showing very variable phenotypes [17,18,19,20,21]. Moreover, it affects a non-conservative aminoacidic residue which may impact on protein structure and/or functions and has a global minor allele frequency of 0.001 (https://www.ncbi.nlm.nih.gov/clinvar/variation/3449/). Monnier et al. reported that this variant is able to affect the signaling activity of the *PROKR2* receptor, thus suggesting its pathogenicity [22]. The pathogenic *GNRHR* variant c.317A>G (p.Gln106Arg) was found in another male subject (Table 1); also this variant has been reported in association with hypogonadotropic hypogonadism [23,24,25]. We identified in another male subject a not reported variant in the *AR* gene causing a premature stop codon and, thus, potentially pathogenic. One female patient carried a pathogenic variant in the *HBB* gene. In addition, *APOB* mutations were identified in 3 female subjects. These findings highlight the diagnostic added-value of One4Two^®^, since it has allowed the detection of pathogenic mutations that should be unveiled by the traditional tests offered to infertile couples.

Finally, we look at some polymorphisms known to be predictive of ovarian stimulation outcomes (Table 2). A meta-analysis, carried out on 57 published studies, evaluated the association between the *FSHR* (rs6165, rs6166, and rs1394205), *LHB* (rs1800447 and rs1056917), and *LHCGR* (rs229327) genes polymorphisms and patients’ clinical outcome, assessed as the number of oocytes retrieved [11]. Interestingly, it emerged that the carriers of the rs6165 in homozygous status had a better outcome (high number of oocytes and shorter stimulation), with respect to the non-carriers or the heterozygous individuals. Similarly, the carriers of the *FSHR* polymorphism rs1394205, both in homozygous or heterozygous status, had a significantly lower consumption of FSH. Even if these data need further studies to assess their application in driving pharmacological choices, however they support the role of specific genotypes in determining reproductive outcome. Thus, since One4Two^®^ want to provide, in a single molecular test, multiple clinically-relevant molecular features to support gynecologists in the planning of the most proper therapeutic approach tailored for each couple, the analysis of the above-mentioned polymorphisms was also included in our method (Table 2). According to previously reported data [11], we are able to identify those women with a better ovarian stimulation outcome based on their genotypes.

The impact of One4Two^®^ on the diagnostic journey of infertile couples was also assessed using an approach based on operation management. From the comparison between the journey of infertile couples before (Figure 4A) and after (Figure 4B) the introduction of One4Two^®^, it is clear that the winning feature of One4Two^®^ is to optimize the entire diagnostic process through the methodological strengthening of the analytical process. The parallelization of analytical operations, by NGS, allows to overcome the bottleneck due to the complexity of managing multiple operations, both for patients (numerous side actions), for diagnostic laboratories (need to use numerous methodologies for perform the required genetic tests), and for reproduction specialists (fragmentary information reported at different times in numerous reports). It has to be underlined that the conception requires the combined and precisely coordinated function of the reproductive system of both partners; as a consequence, the couple evaluation should be mandatory, while it is still unusual in the clinical practice [8].

In addition, the value-added services were analyzed, which in the case of One4Two^®^ are the analysis of genes related to pathologies transmissible to offspring and the analysis of polymorphisms that allow the reproduction specialist to perform controlled ovarian stimulation.

The definition of partners’ carrier status, i.e., couples at risk of transmitting genetic diseases to their offspring, is very important as in most cases these couples do not have a positive family history [5]. For example, for autosomal recessive diseases, heterozygotes are not aware that they are healthy carriers except through genetic testing. The ideal time to assess the partner’s carrier status is before conception, so that preimplantation diagnosis can be considered among the reproductive options [4,26]. Furthermore, the study of *BRCA* mutations gives the opportunity to individualize the path in terms of controlled ovarian stimulation and in vitro fertilization with preimplantation diagnostics options [27]. 

In terms of operation management, the use of NGS allows to reduce the activities not visible to patients, but necessary for the production of analytical data, with a positive impact on the time of the entire process (from 1 year to 1 month), the consumption experience of the infertile couple (as it reduces the time and side actions), and the informative usability of the reproduction specialist. Furthermore, about 20% of infertile couples’ can achieve a successful pregnancy by the correct recognition and treatment of infertility causes [8], thus representing an additional added value of the introduction of One4Two^®^ in routine diagnostic settings.

## 5. Conclusions

The ability of One4Two^®^ to optimize the infertile couples’ journey is linked to the core know-how, represented by the innovative transfer of traditional diagnostic methods to NGS technology. The introduction of One4Two^®^ minimizes the turnaround time, maximizing both the experience of the infertile couples (maximum satisfaction) and of the reproduction specialists (maximum efficiency).

## Figures and Tables

**Table 1:** Pathogenic variants identified in the Training and/or Validation set in genes different from those originally analyzed by traditional diagnostic methods.

Gender	Gene	Reference SNP ID Number (rs)	HGVS ^1^ Coding (cDNA)	HGVS ^1^ Protein Level	Genotype
M	*PROKR2*	rs74315416	c.518T>G	p.Leu173Arg	HET
M	*AR*	nr	c.187delA	p.Gln63fs	HET
M	*GNRHR*	rs104893836	c.317A>G	p.Gln106Arg	HET
F	*APOB*	nr	c.36_38delTGG ^2^	p.Ala13del	HOM
F	*APOB*	rs12713559	c.10672C>T	p.Arg3558Cys	HET
F	*HBB*	rs11549407	c.118C>T	p.Gln40Ter	HET

^1^ Variants nomenclature according to Human Genome Variation Society guidelines; ^2^ Variant identified in 2 independent female subjects; M: male; F: female; HET: heterozygous; HOM: homozygous; nr: not reported.

**Table 2:** Distribution of polymorphisms related to ovarian stimulation outcomes among the 42 women analyzed in the present study.

Gene	Reference SNP ID Number (rs)	HGVS ^1^ Coding (cDNA)	HGVS ^1^ Protein Level	Genotype		
				WT	HET	HOM
*FSHR*	rs6165	c.919G>A	p.Ala307Thr	17	15	10
*FSHR*	rs6166	c.2039G>A	p.Ser680Asn	18	12	12
*FSHR*	rs1394205	c.-29G>A	-	29	12	1
*LHB*	rs1800447	c.82T>C	p.Trp28Arg	39	3	0
*LHB*	rs1056917	c.285T>C	p.Gly95Gly	10	21	11
*LHCGR*	rs2293275	c.935A>G	p.Asn312Ser	8	17	17

^1^ Variants nomenclature according to Human Genome Variation Society guidelines; WT: wild type; HET: heterozygous; HOM: homozygous.

## Supplementary Materials

The following are available online at https://www.mdpi.com/2073-4425/12/1/60/s1, Table S1: List of the genes included in the One4Two® panel.
