# Supplementary material for: One4Two®: An Integrated Molecular Approach to Optimize Infertile Couples’ Journey
Source: Genes (Basel). 2021 Jan 2;12(1):60. doi: 10.3390/genes12010060 (PMC7824287; doi:10.3390/genes12010060)
Supplement: Supplementary file 1 [file genes-12-00060-s001.pdf]

Table S1. List of the genes included in the One4Two® panel. For each gene are reported the mutation rate, the clinical phenotype (with regard to infertility-related issues), and the information provided within the proposed test.

Gene	Mutation Rate*	Phenotype	Information provided
CFTR	0.00003802	- Cystic Fibrosis (Obstructive azoospermia and male infertility)<br>- Congenital Bilateral Absence of the Vas Deferens (CBAVD)	- Genetic cause of infertility<br>- Partners' carrier status
DMD	0.0001005	Duchenne muscular dystrophy	- Partners' carrier status
F5	0.00005807	Thrombophilia	- Genetic cause of infertility
F2	0.00002507	Thrombophilia	- Genetic cause of infertility
F13A1	0.00002477	Thrombophilia	- Genetic cause of infertility
F13B	0.0000162	Thrombophilia	- Genetic cause of infertility
MTHFR	0.00002512	Thrombophilia	- Genetic cause of infertility
SERPINE1	0.00001494	Thrombophilia	- Genetic cause of infertility
ITGB3	0.00002679	Bleeding disorder	- Genetic cause of infertility
FGB	0.0000136	Thrombophilia	- Genetic cause of infertility
APOE	0.00001669	Vascular alterations	- Genetic cause of infertility
APOB	0.0001188	Vascular alterations	- Genetic cause of infertility
ACE	0.00004902	Vascular alterations	- Genetic cause of infertility
FSHR	0.00001783	Ovarian dysgenesis/Ovarian hyperstimulation syndrome	- Genetic cause of infertility<br>- Personalized treatment
GNRHR	0.000008214	Hypogonadotropic hypogonadism	- Genetic cause of infertility
LHB	0.000006597	Hypogonadotropic hypogonadism	- Genetic cause of infertility<br>- Personalized treatment
LHCGR	0.00001819	Hypogonadotropic hypogonadism	- Genetic cause of infertility<br>- Personalized treatment
AR	0.00002504	Androgen insensitivity syndrome/infertility	- Genetic cause of infertility
FSHB	0.000003384	Primary ovarian insufficiency/Hypogonadotropic hypogonadism	- Genetic cause of infertility
INHBA	0.00001418	Ovarian functions alterations	- Genetic cause of infertility
INHBB	0.00001529	Ovarian functions alterations	- Genetic cause of infertility
FMR1	0.00001966	Fragile X/Premature ovarian failure	- Genetic cause of infertility<br>- Partners' carrier status
FMR1NB	0.000009015	Fragile X	- Partners' carrier status
HLA-A	0.00001097	Histocompatibility	- Genetic cause of infertility
HLA-B	0.000009771	Histocompatibility	- Genetic cause of infertility
GALT	0.00001281	Primary ovarian insufficiency	- Genetic cause of infertility
AGT	0.00001559	Thrombophilia/Seminal alterations	- Genetic cause of infertility
FOXL2	0.000008008	Premature ovarian failure	- Genetic cause of infertility
BMP15	0.00001267	Gonadal dysgenesis	- Genetic cause of infertility
ZP1	0.00002076	Oocyte maturation defects	- Genetic cause of infertility
HESX1	0.00000491	Hypogonadotropic hypogonadism	- Genetic cause of infertility
NR5A1	0.00001804	Gonadal dysgenesis	- Genetic cause of infertility
POU1F1	0.000008047	Hypopituarism	- Genetic cause of infertility
SRY	0.000004782	Gonadal dysgenesis	- Genetic cause of infertility
ZFY	0.0000199	Spermatogenic impairment	- Genetic cause of infertility
EIF1AY	0.000002559	Spermatogenic impairment	- Genetic cause of infertility
KAL1	0.0000205	Kallmann syndrome	- Genetic cause of infertility
SF1	0.00002021	Gonadal dysgenesis	- Genetic cause of infertility
DAZ	0.00002663	Spermatogenic impairment	- Genetic cause of infertility
WT1	0.00001554	Gonadal development alterations	- Genetic cause of infertility
FGF8	0.00001017	Hypogonadotropic hypogonadism	- Genetic cause of infertility

FGFR1	0.00003177	Hypogonadotropic hypogonadism	-	Genetic cause of infertility
PROK2	0.000003081	Hypogonadotropic hypogonadism	-	Genetic cause of infertility
PROKR2	0.0000158	Hypogonadotropic hypogonadism	-	Genetic cause of infertility
INSL3	0.000006032	Cryptorchidism	-	Genetic cause of infertility
RXFP2	0.00001967	Cryptorchidism	-	Genetic cause of infertility
CATSPER1	0.00003128	Spermatogenic failure	-	Genetic cause of infertility
HBB	0.000003856	Thalassemia	-	Partners' carrier status
HBA1	0.000003834	Thalassemia	-	Partners' carrier status
HBA2	0.000003017	Thalassemia	-	Partners' carrier status
F8	0.00006063	Haemophilia A	-	Partners' carrier status
F9	0.00001277	Haemophilia B	-	Partners' carrier status
BRCA1	0.00004618	Hereditary breast and ovarian cancers	-	Personalized treatment
BRCA2	0.00008061	Hereditary breast and ovarian cancers	-	Personalized treatment

*evaluated for reported missense variants and based on Varsome database (https://varsome.com/); n.r.: not reported.
